# Disability unplugged: What really matters to people with disabilities?

**DOI:** 10.4102/ajod.v11i0.1172

**Published:** 2022-12-15

**Authors:** Chioma Ohajunwa, Callista Kahonde, Arne H. Eide, Lieketseng Ned

**Affiliations:** 1 Centre for Disability and Rehabilitation Studies, Faculty of Medicine and Health Sciences, Stellenbosch University, Cape Town, South Africa; 2 SINTEF Technology and Society, Oslo, Norway

## Introduction

This special issue presents selected articles that were presented at the sixth African Network for Evidence-to-Action in Disability (AfriNEAD) virtual conference in December 2020. The conference space always affords a reflective space on how far we have come in our quest to drive translation of disability evidence to action. The conference was themed ‘Disability unplugged – Beyond conventions and charters, what really matters to persons with disabilities in Africa’. The theme promoted the presentation of research and community articles as well as a dialogue on issues impacting the day-to-day lives of persons with disabilities beyond the rhetoric of policies and conventions. The articles in this special issue strove to achieve this goal.

The special issue attempted to be inclusive and representative of all the seven conference streams, which can be viewed at https://blogs.sun.ac.za/afrinead/files/2022/05/Updated-Programme-Booklet-2.pdf. A combination of keynote speakers and plenary speakers were approached to submit articles. Initially, a total of 16 articles were expected to be part of this issue; however, not all approached authors were able to submit their articles. All contributions followed a rigorous, blinded peer review process before consideration for publication.

There were 11 articles reviewed in total for this special issue, and 10 of them were accepted for publication after going through the peer review process. The editorial team is therefore delighted to present this special issue comprising the 10 articles (inclusive of original research and opinion articles) that were selected for inclusion. These articles, combined, reflect diversity of research perspectives following the theme of the conference – what really matters to persons with disabilities in Africa?

## An overview of the articles published on this special issue

The first article, written by Luger et al., presents the 9.5 years’ work performed by Chaeli Campaign’s Journal Club, informed by a first-person action research approach. The Journal Club is aimed at strengthening evidence-based practice and contributing to practice-based evidence for children and youth with disabilities in under-resourced South African communities. The authors share their experience of supporting therapists and other community practitioners from under-resourced areas to learn the importance of identifying, evaluating, reading and engaging with available research evidence in a critical manner to inform their practice. In addition, the therapists and community practitioners equally learn how to ethically research, write up and present the interventions they apply at the grassroots level, contributing to a bottom-up and top-down two-way approach of knowledge creation. This rounded approach contributes to locally applicable practice-based evidence, which can influence and encourage other teams to start interdisciplinary journal clubs to support this two-way practice-based evidence.

The second article is written by Ohajunwa, in which the author discusses the outcomes of a study where national inclusive education policies from three countries (South Africa, Ghana and Uganda) were analysed regarding the inclusion of relevant local knowledge within these policies. Informed by a critical, interpretive and constructivist lens, data were gathered through a desktop review of relevant policies and individual key informant interviews. The study revealed a need for more inclusion of local knowledge within inclusive education policies within these contexts. Participants reiterated that the inclusion of local knowledge would better support the implementation of these policies, as there would be increased cohesion between knowledge at home and knowledge in school. Learners with disabilities exist within communities that propagate certain worldviews and ways of being. Having inclusive education policy documents that are responsive to these worldviews, rather than alienating learners, would support more inclusive and sustainable learning outcomes for learners with disabilities.

The third article, authored by Le Roux, discusses the key considerations for facilitating disability-inclusive frameworks that support access to equal opportunities for youth with disabilities in post-apartheid South Africa. Informed by the philosophical frameworks of *ubuntu*, ethics of care and intersectionality, the author draws from an analysis of the experiences of youth from low socio-economic backgrounds who attended a programme at Artscape, in order to explore the attributes of disability inclusion within a post-apartheid South Africa. The author argues that even when they cannot speak, youth with disabilities carry an embodied sense of who they are, which must be acknowledged and respected. Other challenges identified include inadequate physical, technological, social and financial resources and the unaddressed, lingering impact of an apartheid history that continues to negatively impact on their communities. Three main factors that influence access to equal opportunities for these youths emanated from the study. These are enhancement of personal capacity, agency and skills development for youth with disabilities. The author argues that supporting and empowering families to inform sustainable change and effecting systemic and structural changes could facilitate inclusive societies for youth with disabilities.

The fourth article is authored by Vergunst and McKenzie, in which they present a general overview of the work being carried out within the Including Disability in Education in Africa (IDEA) Research Unit at the University of Cape Town in South Africa. In the article, authors highlight that although education is a fundamental right of every child, access to this right is still very much a problem in the Global South. According to the United Nations Educational, Scientific and Cultural Organization (UNESCO), less than 10% of children with disabilities in Africa are in school. The IDEA Research Unit is aligned to a broad vision of inclusive education, informed by a disability studies in education (DSE) approach, positioning disability as a political and social phenomenon, ensuring the voice of people with disabilities and their families are foundational to any related outcomes. The Research Unit’s focus is to provide expert and comprehensive research to inform the paucity of knowledge and decision-making related to disability in education. This is aimed at enhancing the education of children with disabilities and their communities within the context of inclusive educational systems.

In a fifth article, written by Hartley et al., the authors report the outcomes of a longitudinal observational study that was conducted at a rehabilitation centre in the Western Cape, South Africa. The study was focused on a correlation of self-reported health-related quality of life (HRQoL) with activities of daily living (ADL) and stroke severity. Stroke is the third leading cause of disability, and statistics show a global projection of 20 million annual stroke-related deaths and 70 million stroke survivors globally by 2030. Of these, 80% of strokes occur in low- to middle-income countries, with an increased incidence of stroke occurring among younger people in low- to middle-income countries than high-income countries. Sub-Saharan Africa is particularly impacted by this trend. A poststroke survival leaves a heavy financial burden, and it could take years for recovery to happen, with many people not regaining full function. The authors argue that it is critical to support stroke survivors psychologically, emotionally, socially and physically to have a positive perception of their quality of life to inform their well-being.

In the sixth article, written by Visagie et al., the authors present the outcomes of a round-table and small-group discussions on assistive technology. The workshop on collaboration, cohesion and coherence in assistive technology services (ATS) delivery in Africa was part of the AfriNEAD. The authors discuss successful ATS delivery strategies globally and in Africa, stating that although there are some very innovative ATS delivery strategies in Africa, very little is known about these strategies. Despite the presence of various upstream regional and international ATS initiatives, challenges exist. The challenges are linked to policymaking and implementation; inadequate assistive product (AP) provision; poor access to APs and limited data; and uncoordinated and fragmented services, all of which remain very real, daily challenges within the continent. The dominating influence of Western biomedical models over African community approaches, which undermines the capacity of localised services, is raised as a concern. The authors highlight the relevance of end users and communities partnering in ATS delivery and reiterate the importance of research-informed ATS strategies that emanate from the continent, contributing to population health and wellness.

The seventh article, authored by Sadiki, discusses the pivotal role of family in the life of a person with a disability, from the perspective of the author who herself is the mother of a child with a disability, having raised the child in a rural South African context. The author brings up the right of persons with disabilities and their families to equal protection and assistance to enjoy their full rights on an equal basis with others. This is because families and caregivers often provide lifelong support at different levels for the person with a disability. The relevance of early diagnosis and intervention, provision of counselling at the grassroots level within rural communities, working collaboratively with parents to educate them on their rights and building agency aimed at advocacy were highlighted as key areas of focus. Focusing on these areas is identified in the article as imperative to better empower parents and caregivers to continue supporting their family member with a disability.

The eighth article, authored by Van Rensburg-Welling and Mitchell, responds to the challenges faced by training organisations related to lack of training models that accommodate the demands of all learners with disabilities. Informed by a conducted literature review and data from semistructured interviews, the authors propose the adaptable component-based assessment model (ACA) as a potential training model for students with disabilities, which could be assessed to ensure that it is integrated, holistic and student-centred. They argue that the ACA model is an appropriate assessment model as it is based on individual learner affordances, workplace affordances, the holistic development of students and workplace absorption.

The ninth article, authored by Gibberd and Hankwebe, shares transport experiences of people with disabilities during learnerships. These data were retrieved from an evaluative (complaints) questionnaire run by the Department of Transport. While students with disabilities experience challenges related to inaccessible transport, there also seem to be evident tensions between the stipend received versus the transport costs incurred. These shape the participation and, ultimately, completion of learnerships.

The tenth article, authored by Ned, Dube and Swartz, synthesises three keynote presentations delivered at the conference on ‘research evidence’. The focus is particularly on the challenges and opportunities of centring African voices in disability research. The authors argue that the challenges in disability research demand critical scholarship and dedicated activism that help us avoid reproduction and reinforcement of exclusionary practices confronting people with disabilities in Africa.

## Closing remarks

It is our hope that we will see more presentations from the AfriNEAD conference being translated into publication outputs in the near future in order to build African scholarship. We were delighted that, despite coronavirus disease 2019 (COVID-19), this conference was successfully tabled virtually.
